# Evaluation of the prognostic impact of postoperative adjuvant radiotherapy on head and neck mucosal melanoma: a meta-analysis

**DOI:** 10.1186/s12885-015-1750-7

**Published:** 2015-10-21

**Authors:** Wei Li, Yalian Yu, Hailong Wang, Aihui Yan, Xuejun Jiang

**Affiliations:** Department of Otorhinolaryngology, the First Hospital of China Medical University, Shenyang, 110001 People’s Republic of China

**Keywords:** Postoperative radiotherapy, Head and neck mucosal melanoma, Local recurrence, Distant metastasis, Meta-analysis

## Abstract

**Background:**

Head and neck mucosal melanoma (HNMM) is a rare type of malignant tumor that frequently exhibits postoperative recurrence and distant metastasis. Many clinicians administer postoperative adjuvant radiotherapy to improve patient prognosis and enhance quality of life; however, the effects of this treatment remain controversial. Therefore, in this study, a meta-analysis was performed to evaluate the practical value of postoperative adjuvant radiotherapy for head and neck mucosal melanoma.

**Methods:**

Articles in the PubMed, MEDLINE, Cochrane Library, Web of Science and EMBASE databases were systematically retrieved. Analyses were conducted to compare the impact of treatments involving postoperative radiotherapy with treatments entailing surgery alone on patient overall survival time, local recurrence and distant metastasis. The hazard ratio (HR) was used to evaluate the time-to-event data employing RevMan version 5.2 and Stata/SE version 13.0 software according to the principles specified for systematic reviews of interventions in the Cochrane handbook.

**Results:**

Twelve cohort studies involving 1593 patients satisfied the desired conditions. In comparing surgery alone with postoperative radiotherapy, there was no significant difference regarding a decrease in the death risk in HNMM patients (HR, 1.07; 95 % CI, 0.95–1.2; *p* = 0.903; low heterogeneity, I^2^ = 0); this was also the case for sinonasal melanoma after subgroup meta-analysis (HR, 1.04; 95 % CI, 0.8–1.36; *p* = 0.983; low heterogeneity, I^2^ = 0 %). A sensitivity analysis and subgroup meta-analysis showed that disease progression was the main source of the instability in the results. Surgery combined with postoperative radiotherapy reduced the risk of local recurrence (HR, 0.51; 95 % CI, 0.35–0.76; *p* = 0.155) but did not reduce the risk of distant metastasis (HR, 2.26; 95 % CI, 1.01–5.05; *p* = 0.006).

**Conclusions:**

This study demonstrated that for HNMM patients surgery is recommended if indicated, and surgery combined with postoperative radiotherapy is also recommended for dramatically improved local control of the tumor bed. For patients not suitable for surgical treatment, radiotherapy is still advised. To control distant metastasis and finally lower the risk of death, immunological therapy is another potential option whose therapeutic effect needs to be proved with more data from clinical trials.

## Background

Head and neck mucosal melanoma (HNMM) is a rare type of malignant tumor that accounts for 0.8–3.7 % of melanomas [1–5]. The onset of mucosal melanomas is occult; consequently, most patients have late-stage mucosal melanoma by the time the disease is diagnosed. This type of malignant tumor can arise in any mucosal epithelium. The highest incidence is in the head and neck region, which accounts for 55.4 % of all mucosal melanomas, followed by the anus and rectum (23.8 %), the female reproductive tract (18.0 %) and the urinary tract mucosa (2.8 %) [1]. The prognosis for HNMM is poor, with a 5-year overall survival (OS) of 20–33 %. In most cases, lymph node and/or distant metastasis occurs in the early stages of this disease. The 5-year disease-specific survival (DSS) rate for HNMM is 32.4 % [6–8], and the average age range of patients is 55.5–75 years [6–17]. Because of the rarity and invasiveness of HNMM, few relevant studies have examined the diagnosis and treatment of this disease.

At present, there is no uniform staging method for HNMM, and no consensus has been reached regarding treatment regimens for this disease. Surgery is an optional treatment of choice for HNMM. However, the anatomical locations of HNMM present complications, and local and/or distant metastasis has often already occurred when this disease is first diagnosed; therefore, after comprehensively considering the surgical risk and postoperative quality of life, complete resection is extremely difficult to achieve in many cases. Thus, many recent studies have reported on the use of postoperative adjuvant radiotherapy for better local control. However, the prognostic effects of surgery combined with postoperative adjuvant radiotherapy relative to surgery alone, with respect to improving patient survival, remain highly controversial. In addition, chemotherapy and biological therapy have also been used in the adjuvant treatment of HNMM; however, specific therapeutic strategies and their efficacy have not been clearly established.

In the present study, to obtain an unbiased and reliable assessment of the value of postoperative adjuvant radiotherapy for HNMM, a meta-analysis was performed. It involved all publications that met the desired criteria, and compared surgery alone with surgery combined with postoperative radiotherapy. In addition, the study included a systematic review of postoperative radiotherapy treatments and postoperative chemotherapy with or without combined immunobiological therapy. Using these approaches, this study sought to guide the rational development of clinical treatment strategies to improve the survival and quality of life of HNMM patients.

### Statement of translational relevance

This meta-analysis pertains directly to the evaluation of treatment efficacy in oncological clinical trials. We evaluated the relationship among the overall survival time, local recurrence and distant metastasis in HNMM trials. Treatment involving surgery or radiotherapy alone for HNMM could result in a decrease in the death risk. In addition, combination therapy consisting of surgery and radiotherapy could reduce the risk of local recurrence, but showed little effect regarding a reduction in the risk of distant metastasis. No previously published studies have investigated this topic. This 2a level evidence-based analysis demonstrated the strong treatment effects of surgery and radiotherapy in relation to HNMM; more data are needed to elucidate the efficacy of other treatments such as chemotherapy.

## Methods

### Literature retrieval strategy

The systematic computer search involved PubMed, MEDLINE, the Cochrane Library, EMBASE and Web of Science databases (up to December 2014) with the following search strings: “head and neck mucosal melanoma”; “malignant mucosal melanoma”; “sinonasal melanoma”; “nasal melanoma”; “radiotherapy and melanoma”; “chemical therapy and melanoma”; and “biochemical therapy and melanoma”. The language of publication was restricted to English and Chinese. Various referenced publications regarding HNMM, including cohort studies, retrospective clinical studies, reviews and relevant outcome studies, were collected. Supplements involving manual searching for reference lists of all of the searched results were carried out to identify other studies that may have been missed. Publications were screened according to the current study’s inclusion and exclusion criteria.

### Literature inclusion and exclusion criteria

The literature selection process involved the independent retrieval of publications by two different researchers (Wei Li and Yalian Yu). Disagreements were resolved through discussion or by consultation with a third researcher (Hailong Wang). With respect to the literature assessment, for relevant publications, the researchers recorded the authors’ names, addresses and email addresses as well as the literature sources of the publications in question. The authors’ most recent published research was also examined.

### Initial screening criteria

The initial screening criteria were as follows. 1) Study subjects: Patients with a pathological diagnosis of HNMM confirmed by immunohistochemistry who had no other type of malignant tumor. 2) Study type: cohort studies. 3) Intervention measures: A comparison between surgery alone and surgery combined with postoperative radiotherapy; studies involving different surgical procedures and radiotherapy regimens were included for consideration. 4) Result assessment included the following data: (1) OS, which referred to the duration of time from diagnosis or the start of treatment to death, the completion of follow-up, or loss to follow-up; (2) local recurrence (LR), which referred to the duration of time from diagnosis or the start of treatment to the LR of the tumor; (3) distant metastasis (DM), which referred to the duration of time from diagnosis or the start of treatment to the discovery of metastasis in distant organs; and/or (4) adverse reactions related to adjuvant treatment.

### Exclusion criteria

Publications were excluded in accordance with the procedures described by the Cochrane Non-Randomized Studies Methods Group [18]. The titles and abstracts of all retrieved publications were carefully reviewed to exclude publications that clearly did not meet the specified criteria, such as duplicated reports (if reports from different stages of the same study were retrieved, the most recent report was retained for the analyses carried out in the present study), case reports, and reports in which radiotherapy was combined with biological therapy, chemotherapy and/or other types of adjuvant therapies.

The relevant data extracted from the research literature that met the specified criteria were as follows. 1) Literature characteristics (such as the publication’s country or region, authors, title and publication year; the name of the journal in which the study was published; and the authors’ addresses or email addresses). 2) Type of research (for instance, whether a publication was a prospective or a retrospective study). 3) Study elements, including the basic characteristics and disease conditions of the examined cases (for example, the study period, basic information for the enrolled patients, inclusion and exclusion criteria, and duration of follow-up time or time at which patients were lost to follow-up). 4) Interventions (for instance, treatment regimens). 5) Final data that reflected the quality of the research (for example, OS, LR, DM and side effects).

### Assessment of risk of bias regarding the eligible literature

Evaluation criteria: Each publication was independently evaluated by two researchers (Wei Li and Yalian Yu) according to the revised Newcastle-Ottawa Scale (NOS), which was developed to assess non-randomized controlled studies (NRSs) [19]. The between-group comparability of different intervention measures was analyzed using the NOS. We focused on the following factors that affected prognosis: follow-up time; age; primary site (the nasal cavity, a paranasal sinus, the oral cavity, the nasopharynx, or the soft palate among other sites); the surgical approach (endoscopic or open surgery); treatment with postoperative radiotherapy; radiotherapy regimen (a conventional radiotherapy regimen, a proton beam therapy [PT] radiation regimen, or a carbon-ion therapy [CIT] radiation regimen); and the completeness of the final study data (follow-up time and outcome [recurrence, metastasis, survival or death]). The revised evaluation criteria are presented in Table 1.

**Table 1:** Criteria for judgment risk of bias for each study

*Domains*	Judgment criteria for responses to each domain		
	*Yes*	*No*	*Unclear*
*Selection bias*			
*Selection*			
Any criteria descriptions for the patients	Any different radiotherapy plan,tumor stage,local recurrence,distant metastasis,follow duration etc.	Not mentioned	
The representativeness of the postoperative radiotherapy group	truly representative of the average, elderly, community-dwelling resident	somewhat or selected group of patients, e.g. only certain socio-economic groups/areas	no description of the derivation of the cohort
The representativeness of the surgery only group	drawn from the same community as the intervention cohort	drawn from a different source	no description of the derivation of the non intervention cohort
*Comparability*			
Group comparable for:a.average age b.negative margin c patinet status	All the three variables were comparable between the groups	at least one of these was not reported even if others were comparable	Not mentioned
Group comparable for:a.tumor stite b.radiotherapy plan c.tumor stage	All the three variables were comparable between the groups	At least one of those was not comparable even if others were not reported	Not mentioned
Control for confounding at each outcome	Appropriate methods are used to control the potential confounders (e.g. matching, modeling, etc.)	No method was applied to control the potential confounders	Insufficient description
*Design or Analysis bias*			
Blinding of participants at each outcome	1.Blinding of participants at each outcome	1. No blinding or incomplete blinding, the outcome is likely to be influenced by lack of blinding	Insufficient description
	2.No blinding or incomplete blinding, but the reviewers judge that the outcome is not likely to be influenced by lack of blinding	2. Blinding of key study participants and likely that the blinding could been broken, and the outcome is likely to be influenced by lack of blinding	
	3. Blinding of key study participants and unlikely that the blinding could been broken		
Ascertainment of intervention exposure	Medical records or structured interview	Written self report	Insufficient description
*Outcomes*			
Blinding of outcome assessment at each outcome	1. Blinding of outcome assessment at each outcome	1. No blinding of outcome assessment, the outcome measurement is likely to be influenced by lack of blinding.	Insufficient description
	2. No blinding of outcome assessment, but the reviewers judge that the outcome measurement is not likely to be influenced by lack of blinding.		
		2. Blinding of outcome assessment ensured, and likely that the blinding could have been broken and the outcome assessment is likely to be influenced by lack of blinding.	
	3. Blinding of outcome assessment ensured, and unlikely that the blinding could have been broken.		
Ascertainment of outcome data	Record linkage	Self report	Insufficient description
Was follow up long enough for outcomes to occur	The follow-up was long enough for outcomes to occur, if median duration of follow-up > = 6 month	if median duration of follow-up < 6 months	Insufficient description
Adequacy of follow up of cohorts	1. complete follow up: all subjects accounted for	follow up rate < 80 % (select an adequate %) and no description of those lost	Insufficient description
	2. subjects lost to follow up unlikely to introduce bias: number lost < = 20 %, or description of those lost suggesting no different from those followed		

1. Cochrane Handbook for Systematic Reviews of Interventions http://handbook.cochrane.org/. Accessed 2014 Dec 6

2. The Newcastle-Ottawa Scale (NOS) for assessing the quality of nonrandomised studies in meta-analyses: http://www.ohri.ca/programs/clinical_epidemiology/oxford.asp. Accessed 2014 Dec 6

### Data analysis

#### Meta-analysis method

In this study, a quantitative analysis research method was employed. Data analysis and processing were performed using RevMan version 5.2 (The Cochrane Collaboration, Oxford, UK) and Stata/SE version 13.0) software according to the principles specified for Systematic Reviews of Interventions in the Cochrane Handbook.

#### Study effect sizes

The hazard ratio (HR) was used to evaluate the time-to-event data (for example, OS, LR and DM) in treatment efficacy assessments. For documents that did not provide HRs, the HR was calculated using the method described by Tierney et al. [7]. If the HR could not be directly obtained, then the SPSS 17.0 software package was employed to obtain a HR and a 95 % confidence interval (CI) from the raw data (treatment regimen, follow-up time, and endpoint event) using unvaried Cox proportional regression models. If raw data could not be obtained by directly contacting the authors of a publication, the publication in question was excluded from our analysis.

#### Tests of heterogeneity

Heterogeneity was analyzed using chi^2^ tests and I^2^ test. The criterion used for this analysis was a = 0.05; that is, *p* < 0.05 indicated a statistically significant difference. Revman software, version 5.2, was also used to examine heterogeneity using the Peto method. I^2^ was used to represent levels of heterogeneity; in particular, an I^2^ value <25 % indicated low heterogeneity, an I^2^ value of 25–50 % indicated moderate heterogeneity and an I^2^ value of ≥50 % indicated high heterogeneity. If study effect sizes were sufficiently homogeneous, a fixed-effects model was used; if these effect sizes were heterogeneous, a random-effects model was applied.

### Publication bias and sensitivity analyses

Publication bias was estimated using the funnel plot, and quantified by means of the Egger test using Stata/SE version 13.0 software. The heterogeneity may be increased by a number of research methods such as: 1) different features of the study design in the subgroup analyses of different studies, as well as the use of different surgical procedures and/or radiotherapy regimens; and 2) evaluation of potential confounding factors regarding disease progression.

Sensitivity analyses estimated the stability by means of the meta-trim method.

## Results

### Characteristics of the included publications

**Fig. 1:**
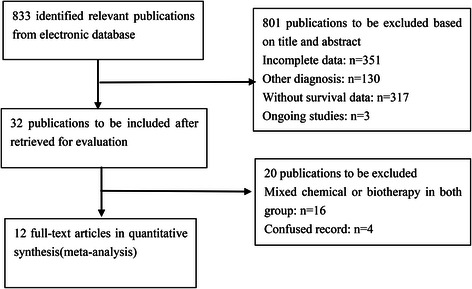
Flow diagram for quantitative studies

Eight hundred and thirty-three records were identified through the literature retrieval strategy initially. After the first screening based on the abstracts and titles, 801 records were excluded, and 32 full texts records remained for further reviewed. An additional 20 records were eventually excluded for 16 of them were mixed chemical or biotherapy in both group and 4 of them were confused records. Ultimately, 12 eligible records were included in this meta analysis. A flow diagram of the study selection is shown in Fig. 1.

A total of 12 investigations (involving 1593 patients) that satisfied the requirements for NRS publications were included in the present study (6, 7, 10–17, 19, 20). These investigations were all cohort studies, and the full text of each of the corresponding publications could be obtained. The relevant information and characteristics of each included publication are presented in Table 2. Both reviewers were in agreement regarding the selection of the 12 studies for the meta-analysis.

**Table 2:** Characteristics of each included publications

Head and neck mucosal melanoma					
Trial & year	Diagnose year	Number	Interventions	Average age(year)^a^	Follow-up(month)
Jethanamest D(2011) [6]	1973-2007	totle815^b^	Surgery only	68.7(17–100)	ungiven
			S + R		
Benlyazid A(2010) [11]	1980-2008	82	Surgery only	67(30–97)	65.2mon
		78	S + R		
Owens JM(2003) [16]	1986-1998	20	Surgery only	55.5(3mon-88y)	ungiven
		24	S + R		
Krengli M(2006) [14]	1972-2002	17	Surgery only	66(40–87)	38(1–207)mon
		42	S + R		
Meleti M(2008) [21]	1976-2006	19	Surgery only	63.7(31–91)	27.8(2–80)mon
		19	S + R		
Temam S(2005) [15]	1979-1997	30	Surgery only	58(21–90)	45.6(8–384)mon
		39	S + R		
Sinonasal melanoma					
Gal TJ(2011) [7]	2000-2007	128	Surgery only	72.1	ungiven
		117	S + R		
Thariat J(2011) [10]	1991-2006	13	Surgery only	73	39(1–181)mon
		10	S + R		
Roth TN(2010) [12]	1992-2007	12	Surgery only	71(40–94)	(7–132)mon
		13	S + R		
Brandwein MS(1997) [17]	1977-1995	17	Surgery only	65(23–83)	59(1–217)mon
		8	S + R		
Cheng YF(2007) [13]	1982-2002	totle23^b^	Surgery only	68.2(35–87)	3-132mon
			S + R		
Sun CZ(2014) [20]	1976-2005	18	Surgery only	55(2–79)	6-114mon
		13	S + R		

S + R:postoperative radiotherapy

^a^Age at first diagnosis

^b^studies that cannot separate the surgery only group from the postoperative radiotherapy group in number

Among the included publications, there were five multicenter studies [6, 7, 11, 14, 15] and seven single-institution non-multicenter studies [10, 12, 13, 16, 17, 19, 20]. In cases of HNMM, the primary tumor was located in various sites of the head and neck mucosa, such as the nasal cavity, paranasal sinus, nasopharynx, oral cavity or oropharynx, among other locations [1]. Cases of paranasal sinus or nasal mucosal melanoma (SNMM) account for the majority (72.2 %) of HNMM cases and have similar characteristics to other mucosal melanomas [19]; consequently, publications examining mucosal melanomas of the nasal cavity and the paranasal sinuses were also included in this study. Thus, the included publications consisted of six HNMM studies [6, 11, 14–16, 19] and six SNMM studies [7, 10, 12, 13, 17, 20]. High intervention variability among NRSs is inevitable. Moreover, there is no uniform treatment strategy for HNMM patients. In the postoperative radiotherapy group, most investigators used either traditional radiotherapy with conventional radiation doses [19], low-dose fractionated radiotherapy [10] or mixed radiotherapy (in which certain patients received conventional doses and the remaining patients received low-dose fractionated radiotherapy, but the authors did not provide additional specifics) [11, 13, 14, 20]; other publications did not specifically report their radiotherapy strategies [6, 7, 11, 12, 19]. Descriptive evaluations of the related bias of the included publications are presented in Table 3. Eligible data could not be retrieved from all of the included publications. Therefore, in the final meta-analysis, 12 publications were used for OS analysis [6, 7, 10–13, 15–17, 19, 20], five publications matched the criteria for local control analysis [11, 12, 14, 16, 17] and four publications matched the criteria for DM analysis [11, 12, 16, 17].

**Table 3:** Assessment of risk of bias of included cohort studies

Criteria	Jethanamest D	Benlyazid A	Owens JM	Krengli M	Meleti M	Temam S
	(2011) [6]	(2010) [11]	(2003) [16]	(2006) [14]	(2008) [21]	(2005) [15]
Any criteria descriptions for the patients	yes	yes	yes	yes	yes	yes
The representativeness of the surgery only group	yes	yes	yes	yes	yes	yes
The representativeness of the postoperative radiotherapy group	yes	yes	yes	yes	yes	yes
Group comparable for:	yes	yes	yes	yes	yes	yes
a.average age b.negative margin c patinet status						
Group comparable for:	unclear	unclear	unclear	unclear	unclear	unclear
a.tumor site b.radiotherapy plan c.tumor stage						
Control for confounding at each outcome(OS)^a^	yes	yes	no	unclear	no	unclear
Control for confounding at each outcome(LR)^a^	unclear	yes	no	yes	no	unclear
Control for confounding at each outcome(DM)^a^	unclear	yes	no	unclear	no	unclear
Blinding of participants at each outcome	yes	yes	yes	yes	yes	yes
Ascertainment of intervention exposure	yes	yes	yes	yes	yes	yes
Blinding of outcome assessment at each outcome	yes	yes	yes	yes	yes	yes
Ascertainment of outcome data	yes	yes	yes	yes	yes	yes
Was follow up long enough for outcomes to occur(OS)	yes	yes	yes	no	yes	yes
Was follow up long enough for outcomes to occur(LR)	no	yes	yes	yes	no	yes
Was follow up long enough for outcomes to occur(DM)	no	yes	yes	no	no	yes
Adequacy of follow up of cohorts	unclear	unclear	unclear	unclear	unclear	unclear
Criteria	Gal TJ	Thariat J	Roth TN	Brandwein MS	Cheng YF	Sun CZ
	(2011) [7]	(2011) [10]	(2010) [12]	(1997) [17]	(2007) [13]	(2014) [20]
Any criteria descriptions for the patients	yes	yes	yes	yes	yes	yes
The representativeness of the surgery only group	yes	yes	yes	yes	yes	yes
The representativeness of the postoperative radiotherapy group	yes	yes	yes	yes	yes	yes
Group comparable for:	yes	yes	yes	yes	yes	yes
a.average age b.negative margin c patinet status						
Group comparable for:	unclear	unclear	unclear	unclear	unclear	yes
a.tumor site b.radiotherapy plan c.tumor stage						
Control for confounding at each outcome(OS)^a^	yes	no	no	no	no	yes
Control for confounding at each outcome(LR)^a^	unclear	no	no	no	no	unclear
Control for confounding at each outcome(DM)^a^	unclear	no	no	no	no	unclear
Blinding of participants at each outcome	yes	yes	yes	yes	yes	yes
Ascertainment of intervention exposure	yes	yes	yes	yes	yes	yes
Blinding of outcome assessment at each outcome	yes	yes	yes	yes	yes	yes
Ascertainment of outcome data	yes	yes	yes	yes	yes	yes
Was follow up long enough for outcomes to occur(OS)	yes	yes	yes	yes	yes	yes
Was follow up long enough for outcomes to occur(LR)	no	yes	yes	yes	yes	no
Was follow up long enough for outcomes to occur(DM)	no	yes	yes	yes	yes	no
Adequacy of follow up of cohorts	unclear	unclear	unclear	unclear	unclear	unclear

*OS* overall survival time, *LR* local recurrence, *DM* distance metastasis

^a^confounding factors means other intervening measures that may effect the outcomes(eg. chemical therapy etc.)

### Survival data

**Fig. 2:**
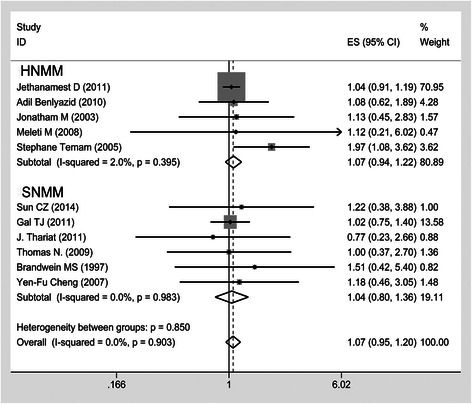
Forest plots of postoperative radiotherapy vs. surgery alone group on the overall survival time in HNMM by the subgroup analysis for SNMM

**Fig. 3:**
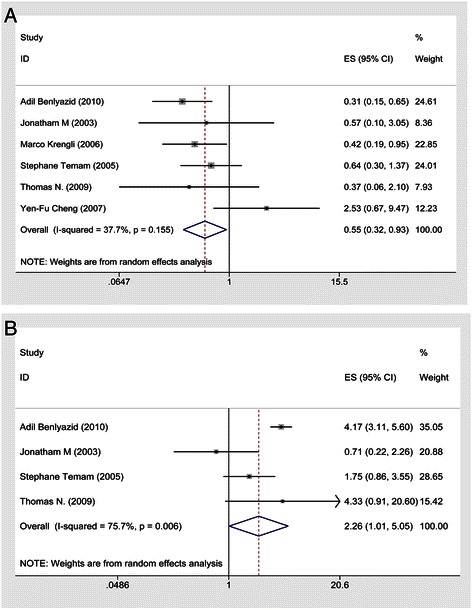
**a** Forest plots of postoperative radiotherapy vs. surgery alone group on the LR for HNMM; (**b**) Forest plots of postoperative radiotherapy vs. surgery alone group on the DM for HNMM

There was no significance difference between surgery alone and postoperative radiotherapy regarding a decrease in the death risk; in 11 cohort studies involving 1565 patients, the HR was 1.07, the 95 % CI was 0.95–1.20 and there was low heterogeneity (I^2^ = 0). Furthermore, postoperative radiotherapy did not reduce the risk of death among SNMM patients (HR, 1.04; 95 % CI, 0.80–1.36; heterogeneity, *p* = 0.983, I^2^ = 0 %) in fixed-effects models (Fig. 2). However, the subgroup meta-analyses of the LR demonstrated that postoperative radiotherapy could reduce the risk of local recurrence by 45 % in a random-effects model (six cohort studies involving 382 patients [HR, 0.55; 95 % CI, 0.32–0.93; heterogeneity, I^2^ = 38 %; *p* = 0.15; Fig. 3a). In addition, the random-effects model for the subgroup meta-analysis of DM revealed that postoperative radiotherapy did not reduce the risk of DM (HR, 2.26; 95 % CI, 1.01–5.05; heterogeneity, I^2^ = 76 %; *p* = 0.006; Fig. 3b).

**Fig. 4:**
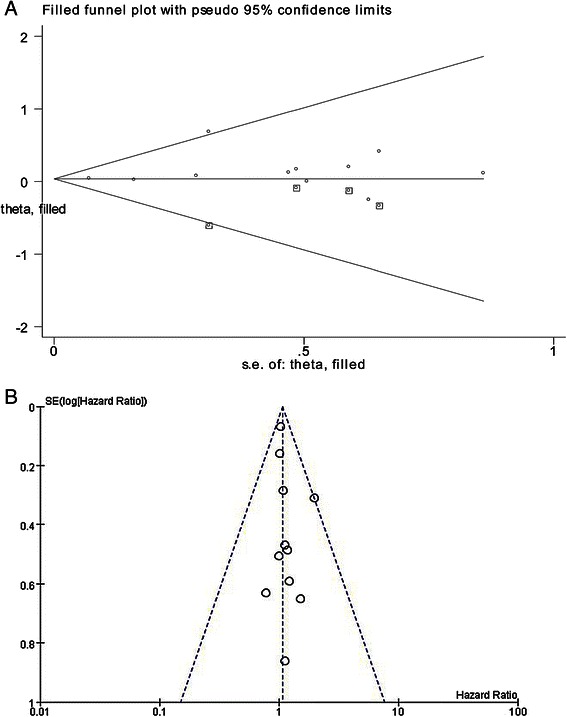
**a** Funnel plot analysis of Sensitivity analyses for included studies; (**b**) Funnel plot analysis of publication bias for included studies

Sensitivity analyses estimated stability using the meta-trim method (Fig. 4a); after evaluating three studies, our new meta-analysis gave the same result [HR, 1.037; 95 % CI, 0.927–1.160; *p* = 0.524). This proved the stability of the conclusion, namely that postoperative radiotherapy could reduce the risk of death. Publication bias was tested using the funnel plot (Fig. 4b). The Egger test used to quantify the publication bias (*p* = 0.290) showed no small-study effects, which confirmed the results did not reveal an obvious asymmetry.

## Discussion

Twelve retrospective studies that examined a total of 1593 patients were included in the current investigation. All of these studies were descriptive cohort studies, and they provided moderately strong evidence. Given that HNMM is rare and is associated with an extremely poor prognosis, the results of this study can nonetheless provide significant guidance in making clinical treatment decisions regarding this disease.

### The preferred treatment method: surgical resection

The subjects included in the current study were patients with a history of past surgical treatment; for mucosal melanoma surgery achieves better results than other therapeutic approaches with respect to treatment and local control. Moreover, surgery was the first treatment for many patients in the vast majority of examined publications. In an examination of 815 HNMM patients, Thomas et al. [6] found that compared with patients who received surgery alone, the relative risk ratios for patients who received simple radiotherapy or no treatment were 1.56 (95 % CI, 1.35–1.72) and 2.38 (95 % CI, 2.09–2.59), respectively. The clinical significance of these findings was that compared with the administration of surgical treatment, the administration of radiotherapy alone and the administration of no treatment increased the risk of death by 56 and 138 %, respectively. Thus, patients who underwent surgical treatment (with or without postoperative radiotherapy) had better prognoses and longer survival times than patients who did not receive surgical treatment. In a study by Andersen et al., all surviving patients had received surgical treatment; however, the non-surgical treatment approach involving chemoradiotherapy alone had little or no therapeutic effect [5].

Furthermore, the 2012 National Comprehensive Cancer Network (NCCN, an American organization) guidelines recommend surgery as the preferred treatment for mucosal melanoma patients; in particular, the optimal objective for surgical treatment is en bloc resection that effectively achieves tumor-free margins (NCCN Guidelines Version 1.2012, Mucosal Melanoma of the Head and Neck). However, surgical treatment is only suitable for patients with stage III–IVA cancer in the American Joint Committee on Cancer (AJCC) staging system (7th edition). In these stages, cancer is limited to the mucosal layer or exhibits moderate progression; that is, the tumor may have invaded deep soft tissues, cartilage, bone or surface skin covering the tumor, but there is no lymph node metastasis or DM [19].

Unfortunately, most HNMM patients are already at an advanced clinical and pathological stage of the disease when they are first diagnosed; as a result, their prognoses are far worse than the prognoses of cutaneous melanoma patients, for whom surgical treatment is also the preferred therapeutic approach [21, 22]. When cutaneous melanoma patients are first diagnosed, the percentage of patients at stages 0, I, II, III, and IV (using AJCC staging) are 14.9, 47.7, 23.1, 8.9 and 5.3 %, respectively; many patients undergo surgery or other effective interventions at the early stages of this disease. A survey of 84,836 melanoma patients indicated that 91.5 % of cutaneous melanoma patients underwent surgery, and that the 5-year survival rate for cutaneous melanoma was 80.8 % [1]. However, for HNMM, because of complexities associated with the anatomical location of the primary tumor, it is often difficult to achieve negative safety margins during the microscopic resection of this tumor [4]. In particular, it is very difficult to completely resect paranasal sinus mucosal melanomas that are located near the cribriform plate or the skull plate, or that have invaded the anterior skull base [23]. Therefore, many researchers believe that it is unwise to attempt to achieve complete resection at the expense of increasing surgical risk and reducing postoperative quality of life [24, 25]. However, a lack of complete resection increases postoperative complications and thereby affects patient prognosis.

### Postoperative radiotherapy is effective for local control, but does not reduce the risks of DM and death

In the present study, a random-effects model was used for the meta-analysis of the effects of postoperative radiotherapy on the local control (assessed in terms of LR) of HNMM. Relative to surgery alone, surgery combined with postoperative radiotherapy can reduce the risk of LR by 45 % (five NRSs with a total of 336 patients; HR, 0.55; 95 % CI, 0.32–0.93; *p* < 0.05). However, postoperative radiotherapy did not reduce the risk of postoperative death in HNMM patients (nine NRSs with a total of 1465 patients; HR, 1.07; 95 % CI, 0.95–1.02). Postoperative radiotherapy also did not reduce the risk of death in cases of SNMM (five NRSs with a total of 341 patients; HR, 1.04; 95 % CI, 0.79–1.36).

Because HNMM is multicentric, the clinical boundaries of this disease are blurred and unclear; infiltration occurs, particularly submucosal lymphoid infiltration involving melanoma cells [26], resulting in a high rate of postoperative LR (31–85 %) [27]. Therefore, even if HNMM is diagnosed early and aggressive surgical treatment is administered, postoperative adjuvant treatment remains necessary. Among the possible adjuvant treatments, postoperative radiotherapy is one of the earliest examined adjuvant therapies; it has been used in a relatively large number of clinical cases. The purpose of radiotherapy is to reduce the postoperative invasion of residual tumors into the surrounding normal tissues. The NCCN has noted that postoperative radiotherapy is suitable in cases with extra capsular lesions, the invasion of two or more neck or parotid gland lymph nodes, a single nodule ≥3 cm and neck dissection without distant invasion or postoperative LR. The use of conventional radiotherapy doses (2 Gy per fraction, at a total dose of 60–66 Gy or 70 Gy) is recommended. In addition, secondary damage from radiotherapy is regarded as acceptable. The current study confirmed that although the adjuvant treatment approach outlined above produced good local control effects, it did not reduce the risk of death. The reasons for this phenomenon are analyzed below.

First, HNMM is a radiotherapy-resistant tumor; that is, HNMM tumors exhibit extremely strong sublethal damage repair capacities. Therefore, although conventional radiotherapy doses can achieve local control, traditional radiotherapy is ineffective in controlling the disease as a whole. For this reason, stereotactic intensity-modulated radiation therapy (IMRT), PT and CIT have recently been developed for the adjuvant treatment of HNMM. Although these novel approaches have exhibited enhanced efficacy relative to conventional radiotherapy, they remain at the clinical trial stage; thus, numerous clinical trials and prospective studies will be required to determine the long-term efficacy of these treatments.

Second, mucosal melanoma is a systemic disease that readily invades vascular and lymphoid tissues and therefore characteristically tends to produce DM. Thus, it is difficult for local radiotherapy to achieve good control of the hematogenous metastasis of this disease [24]. Notably, many mucosal melanoma patients die from the spread of the disease rather than from LR. Rinaldo et al. [27] have demonstrated that in contrast to primary squamous cell carcinoma of the nasal cavity and the paranasal sinuses, which mainly exhibits posterior pharyngeal wall and mandibular lymph node metastasis, mucosal melanoma more frequently exhibits lung and brain metastasis [28]. Consequently, although postoperative adjuvant radiotherapy has relatively strong local control effects, it is not a protective factor for HNMM because the main cause of death in certain HNMM patients is DM.

Finally, because of the rarity of HNMM, it is extremely difficult to conduct large-sample RCTs for the examination of this disease. Therefore, selection bias in the meta-analysis of cohort studies will have caused uncertainty in the results of the present study. In other words, in the included retrospective studies, most patients who received postoperative radiotherapy were late-stage cases involving failed en bloc resection, positive safety margins, lymphatic vascular invasion and/or DM. However, it was determined that for these patients, postoperative radiotherapy could still achieve local control but did not reduce the risk of death.

### Value of other treatment methods in non-surgical therapy

As discussed above, surgical treatment is difficult to implement in HNMM cases because of various issues including: complications associated with the anatomical location of a tumor (particularly in the case of SNMMs located near important organs); the advanced stage of the disease upon initial diagnosis; patient refusal of surgical treatment; and the comprehensive consideration of surgical risk and postoperative quality of life. Consequently, to increase survival rates and prolong patient survival times in HNMM cases, many researchers advocate the use of radiotherapy, chemotherapy and/or biological therapy alone as alternatives to surgical treatment.

### Evaluation of the therapeutic efficacy of simple radiotherapy for HNMM

In recent years, with the continuous advancement of radiotherapy techniques and the upgrading of relevant equipment, clinicians have gradually begun to increase the use of radiotherapy alone in the treatment of primary HNMM. Although the development of specific therapeutic strategies remains controversial, studies have already proven the efficacy of this approach and have demonstrated that the side effects of the radiotherapy are within manageable ranges.

Douglas et al. [29] recommended radiotherapy as the preferred treatment approach for HNMM patients who cannot withstand surgery or refuse surgical treatment. In a retrospective study of 68 HNMM patients, 13 patients received palliative radiotherapy, 30 received radiotherapy at therapeutic doses and 25 received surgical treatment with or without postoperative radiotherapy. Radical treatment mainly using radiotherapy achieved relatively good local control effects and a 5-year DSS of 25 %. However, there have been few relevant reports on the use of radiotherapy at therapeutic doses, and these reports have involved small sample sizes. These limitations occurred because HNMM is a radiotherapy-resistant tumor with extremely effective sublethal repair capabilities. Therefore, therapeutic hypofractionated radiotherapy (HF-RT) with an α/β ratio of 4–6 Gy per fraction has been used to overcome this radiotherapy resistance. The basic dose for the treatment of uveal melanomas is 1.5–26.2 Gy, with an average of 10 Gy, and the basic dose for cutaneous melanomas is 1.6–6 Gy, with an average of 2.5 Gy; however, the dose for mucosal melanoma remains unknown [30]. Wada et al. [31] performed high-dose HF-RT with doses ≥3 Gy in 31 HNMM patients. They found that compared with conventional postoperative radiotherapy, HF-RT delivered better local control and survival; in particular, HF-RT resulted in 1- and 3-year DSS rates of 73 and 33 %, respectively. Moreover, increased radiotherapy doses produced better therapeutic effects with respect to local control and survival. However, with the aforementioned radiotherapy regimen, the occasional occurrence of lethal side effects, such as mucosal ulceration and massive hemorrhage, is inevitable. Christopherson et al. [32] administered therapeutic doses of radiotherapy or postoperative radiotherapy to 21 patients. They found that three patients (17 %) experienced serious complications, including the need for hospitalization and/or surgical intervention, failing to complete the entire radiotherapy regimen, and death. Two of these patients received PT alone, and one patient received photon therapy and PT. The manifestations of the complications included bilateral blindness, severe mucositis and skin necrosis. Thus, numerous clinical studies involving HF-RT are still required to develop regimens with optimal therapeutic effects and ensure that the side effects of these regimens are manageable.

Given the side effects of HF-RT, local dose-escalated IMRT, another improved photon radiotherapy technique, has been used in clinical practice. In a study on the treatment of malignant tumors of the paranasal sinuses, Madani et al. [33] reported that IMRT was the gold standard for radiotherapy approaches for these tumors, and that IMRT could be used alone or as an adjuvant treatment. Combs et al. [34] used IMRT to treat eight SNMM patients and found a 5-year OS of 80 %, a 3-year local progression-free survival rate of 28.6 % and a 3-year distant progression-free survival rate of 28.6 %. In addition, during the 27 months (12–71 months) of follow-up, radiotherapy-related side effects were within acceptable ranges. No vision loss or blindness occurred, and additional therapeutic intervention was not required. However, both the aforementioned HF-RT approach and this IMRT approach produced non-ideal effects with respect to the control of DM.

At present, PT and CIT particle therapies achieve better local control of melanomas than surgery alone, conventional photon radiotherapy, or a combination of surgery and photon radiotherapy. Therefore, the use of PT or CIT particle radiotherapy alone for HNMM is attracting widespread attention. Because PT and CIT can provide precisely distributed radiation doses, these techniques can be used to specifically deliver high doses of radiation to tumors with a reduced risk of radiation exposure to normal tissues; this characteristic is particularly relevant for malignant tumors located in or near important tissues and organs [28, 35]. In contrast to the high levels of attenuation characteristic of the traditional photon beams used for radiotherapy upon penetrating soft tissues, proton beams exhibit tremendous energy deposition in targeted tumors but minimal energy deposition in the preceding or subsequent normal tissues; consequently, proton beams can be used to accurately deliver radiation to tumors with relatively little effect on the surrounding normal tissues [36, 37]. This characteristic is highly suitable for the treatment of paranasal sinus melanomas because the radiotherapy dose required for the treatment of these tumors is extremely similar to the doses that affect the surrounding normal tissues, such as the eyes, the optic nerves, brain tissues and the skull. Compared with photon beam radiotherapy, PT is associated with lower risks regarding the development of secondary malignant tumors [33, 34]. In an examination of 11 cases treated with PT, the initial disease control rate was 85.7 %, the 3-year survival rate was 58.0 % and the mean disease-free time to progression was 25.1 months (with a mean follow-up time of 36.7 months); there were two cases of unilateral vision loss but no blindness events [38]. In addition, in 72 cases treated using CIT, the 5-year local control rate for the disease was 84.1 %; 94.4 % of the patients who exhibited good local control received radiotherapy with ≥3.6 Gy/fraction. However, by the end of the follow-up, DM had occurred in 40 cases; LR had not occurred in 34 of these cases (85.0 %). These results confirmed that the tumors had often already invaded their microenvironment before treatment had begun and that the examined radiotherapy approach did not produce good control of HNMM that underwent early-stage hematogenous DM [39]. In contrast, compared with proton beams, carbon-ion beams have a better penumbra and higher relative biological effectiveness. However, Demizu et al. [40] demonstrated that PT and CIT did not exhibit significant differences regarding therapeutic effectiveness in the treatment of HNMM; in particular, the 2-year survival rates among patients treated with PT and CIT were 58 and 62 %, respectively (*p* = 0.399; Kaplan-Meier [K-M] curve analysis). PT and CIT also had no significant differences regarding local control effects; these treatments resulted in local control rates of 83 and 59 %, respectively (*p* = 0.569; K-M curve analysis). In addition, the radiotherapy side effects of PT and CIT did not differ significantly. Although CIT and PT particle therapies had excellent local control effects (with an effective 2-year local control rate of 78 %), their outcomes regarding OS were not ideal (a 2-year DSS of 31 %). The analysis appeared to indicate that this phenomenon could be attributed to a relatively high incidence of DM. Thus, although the toxic side effects of radiotherapy could be controlled to within tolerable levels, the effects of particle radiotherapy with respect to controlling DM were not ideal.

In summary, the value of radiotherapy in the treatment of HNMM should receive extensive attention in future studies. With the advancement of radiotherapy techniques and constant upgrading of the relevant equipment, solutions that address the issue of radiotherapy side effects may be developed in the near future. Numerous controlled clinical studies and prospective studies are still required to establish radiotherapy regimens that achieve therapeutic objectives, but produce manageable side effects. In addition, because mucosal melanoma is a systemic disease, therapeutic approaches that combine radiotherapy with chemotherapy or immunobiological therapy can be designed to compensate for the deficiencies of radiotherapy alone, with respect to controlling the high incidence of DM in cases of mucosal melanoma.

### Immunobiological therapy

Compared with cutaneous melanoma, mucosal melanoma involves more chromosomal abnormalities and abnormal gene copies [41]. At present, gene therapies targeting the mutation hotspots of malignant melanoma have already entered clinical trials. In particular, a phase III clinical trial has confirmed that vemurafenib, a selective BRAF inhibitor, produces a higher response rate (48 % vs 5 %) and 6-month OS (84 % vs 64 %) compared with decarbonizes, another systemic chemotherapeutic agent [42]. In a phase II clinical trial, imatinib, a targeted therapeutic agent for melanomas designed to be effective in cases involving KIT mutations, exhibited outstanding performance; it achieved a significantly higher response rate in melanomas with KIT mutations than in wild-type melanomas (40 % vs 0 %; *p* = 0.005) [43]. A recent phase II clinical trial confirmed that patients with NRAS (neuroblastoma RAS viral [v-ras] oncogene homolog) mutations could benefit from treatment with MEK1/2 inhibitors (MAP/ERK kinases 1 and 2) [44]. The mutation rates of KIT, NRAS, and BRAF mutations among SNMM patients were 7.91 % (11/139), 12.9 % (18/139) and 2.16 % (3/139), respectively [43, 45–48]. Analysis of the above data revealed that there are relatively low rates of KIT and BRAF mutations among SNMM patients. Consequently, few SNMM patients will benefit from chemotherapeutic drugs that target these two genes.

Rosenberg et al. [49] demonstrated that the transfer of lymphokine-activated killer (LAK) cells induced by interleukin-2 (IL-2) could increase the survival rate of patients with malignant tumors. In addition, mucosal melanoma is a systemic disease that readily undergoes DM; thus, LAK immunobiological therapy can help to improve the prognosis of mucosal melanoma patients. LAK cellular therapy is an emerging treatment modality that has been applied to mucosal melanoma in recent years [49, 50–52]. The core steps in this treatment are the collection of venous blood, the *in vitro* incubation of these blood samples with a certain concentration of IL-2, and the subsequent transfusion of the blood back into patients; patients are then treated with sustained administration of an appropriate concentration of IL-2 to maintain LAK activity [52, 53]. Kanetaka et al. [54] examined postoperative treatment with IL-2 and LAK in 13 HNMM patients and found that the 5-year DSS rates of the group receiving the immunobiological treatment and the untreated group were 67 and 33 %, respectively. DM is regarded as an important cause of death among HNMM patients. Although surgery, radiotherapy and chemotherapy are the most common treatment approaches, they do not produce particularly strong effects with respect to controlling DM. Thus, although biological therapy for HNMM is uncommon, this type of treatment approach nonetheless has potential research value as an adjuvant therapy.

Moreover, in recent years, it has been confirmed that immunobiological therapy produces certain radiation-sensitizing effects on radiotherapy-resistant tumors. The local tissue hypoxia caused by the strong metabolic activity of tumor cells can induce local immunosuppressive effects, which are manifested as tumor-promoting disruptions in normal innate immunity [19]. Tissue hypoxia can induce the local downregulation of the interferon-γ (IFN-γ) immune response pathway and thereby inhibit the activation of various mediating factors, such as CD4+/CD25+ regulatory factors (where CD represents a cluster of differentiation), IL-10 and transforming growth factor-b. A study has demonstrated that the phenotype of activated IFN + CD8+ T cells was associated with a 1.8-fold increase in tumor sensitivity to radiotherapy [55]. Therefore, IFN-γ + CD8+ T cells serve as a bridge between immune and radiotherapy responses, and can produce targeted radiosensitization effects. Clinically available immunological adjuvants and tumor vaccines can become effective tools to simultaneously stimulate tumor immunosurveillance and enhance radiotherapy responses. Although HNMM is highly malignant, has a low incidence, and occurs through molecular mechanisms that have not yet been fully elucidated, there are nonetheless broad prospects for the potential use of biological therapies in the treatment of this disease.

### Study limitations

#### Potential bias in the included NRSs

Greater potential biases exist in NRSs than in RCTs [18] Among these biases, selection bias is a major concern because factors that influence prognosis occurred unevenly across different groups in the selected studies. In addition, commonly observed imperfections in the NRSs remain present in the reports, and in descriptions of research protocols and the evaluations of measurements that affect prognoses and results. These flaws all contribute to uncertainties in meta-analyses. In particular, because a meta-analysis is a non-experimental observation study, completely uniform control standards could not be achieved in the current meta-analysis; one specific manifestation of this issue is that uniform quality control cannot be performed in each case. Therefore, subgroup analysis (such as separate examinations of different radiotherapy regimens, different clinical and case stages, and cases involving lymphatic vascular invasion) cannot be performed. These issues caused selection bias in evaluations regarding the efficacy of postoperative adjuvant radiotherapy, because in the included retrospective studies postoperative radiotherapy was mostly used in patients with a positive safety margin, local metastasis, DM and/or primary tumors with larger scopes.

#### Side effects of radiotherapy

HNMM lesions are located near various important tissues and organs, such as the optic nerves and brain tissues. Therefore, during the course of surgery and radiotherapy patients may experience various adverse effects, including optic nerve damage and mucositis, which will affect prognosis. However, the relevant data of the included publications were incomplete, and these publications did not use uniform quality control standards; consequently, the prognostic impact of radiotherapy-related side effects on prognosis was not analyzed. However, relevant reports have indicated that certain side effects affect patient prognoses and survival times.

#### Radiotherapy subgroup analysis

In the present study the influence of radiotherapy on prognosis, local control and metastasis was analyzed. However, recent advances in radiotherapy techniques and upgrades to radiotherapy equipment have led to the development of novel radiotherapy strategies, such as PT and CIT. In the context of HNMM, these novel strategies are superior to conventional radiotherapy because they can both control LR and improve patient survival. In addition, compared with conventional radiotherapy, these emerging radiotherapy approaches clearly produce reduced side effects. However, there are few relevant reports regarding these approaches, the extant reports involve small sample sizes, and studies of many potential novel radiotherapy regimens remain in the experimental stage. Therefore, future research will be required to address this topic.

## Conclusions

The current meta-analysis confirmed that the use of postoperative radiotherapy can improve the local control of HNMM but does not reduce the risk of death or distant metastasis. However, with the development of molecular mechanisms there are nonetheless broad research prospects for the use of biological therapy and radiotherapy in the treatment of this disease. Determination of the long-term effects of other adjuvant therapies on patient prognosis will require additional examination involving a large number of prospective clinical and experimental studies.

## Abbreviations

CIs: Confidence intervals
CIT: Carbon-ion therapy
DM: Distant metastasis
DSS: disease-specific survival
HF-RT: Therapeutic hypo-fractionated radiotherapy
HNMM: Head and neck mucosal melanoma
HR: Hazard ratio
IL-2: Interleukin-2
IMRT: Intensity-modulated radiation therapy
LAK: Lymphokine-activated killer
LR: Local recurrence
NOS: Newcastle-Ottawa Scale
NRSs: Non-randomized controlled studies
OS: Overall survival
PT: Proton beam therapy
SNMM: Sinus or nasal mucosal melanoma

## References

[1] Chang AE, Karnell LH, Menck HR (1998). The national cancer data base report on cutaneous and noncutaneous melanoma: a summary of 84,836 cases from the past decade. The American college of surgeons commission on cancer and the american cancer society. Cancer.

[2] Patrick RJ, Fenske NA, Messina JL (2007). Primary mucosal melanoma. J Am Acad Dermatol.

[3] Wong JH, Cagle LA, Storm FK, Morton DL (1987). Natural history of surgically treated mucosal melanoma. Am J Surg.

[4] Manolidis S, Donald PJ (1997). Malignant mucosal melanoma of the head and neck: review of the literature and report of 14 patients. Cancer.

[5] Andersen LJ, Berthelsen A, Hansen HS (1992). Malignant melanoma of the upper respiratory tract and the oral cavity. J Otolaryngol.

[6] Jethanamest D, Vila PM, Sikora AG, Morris LG (2011). Predictors of survival in mucosal melanoma of the head and neck. Ann Surg Oncol.

[7] Gal TJ, Silver N, Huang B (2011). Demographics and treatment trends in sinonasal mucosal melanoma. Laryngoscope.

[8] Wu AJ, Gomez J, Zhung JE, Chan K, Gomez DR, Wolden SL (2010). Radiotherapy after surgical resection for head and neck mucosal melanoma. Am J Clin Oncol.

[9] Moreno MA, Roberts DB, Kupferman ME, DeMonte F, El-Naggar AK, Williams M (2010). Mucosal melanoma of the nose and paranasal sinuses, a contemporary experience from the M. D. Anderson Cancer Center. Cancer.

[10] Thariat J, Poissonnet G, Marcy PY, Lattes L, Butori C, Guevara N (2011). Effect of surgical modality and hypofractionated split-course radiotherapy on local control and survival from sinonasal mucosal melanoma. Clin Oncol.

[11] Benlyazid A, Thariat J, Temam S, Malard O, Florescu C, Choussy O (2010). Postoperative radiotherapy in head and neck mucosal melanoma: a GETTEC study. Arch Otolaryngol Head Neck Surg.

[12] Roth TN, Gengler C, Huber GF, Holzmann D (2010). Outcome of sinonasal melanoma: clinical experience and review of the literature. Head Neck.

[13] Cheng YF, Lai CC, Ho CY, Shu CH, Lin CZ (2007). Toward a better understanding of sinonasal mucosal melanoma: clinical review of 23 cases. J Chin Med Assoc.

[14] Krengli M, Masini L, Kaanders JH, Maingon P, Oei SB, Zouhair A (2006). Radiotherapy in the treatment of mucosal melanoma of the upper aerodigestive tract: analysis of 74 cases. A rare cancer network study. Int J Radiat Oncol Biol Phys.

[15] Temam S, Mamelle G, Marandas P, Wibault P, Avril MF, Janot F (2005). Postoperative radiotherapy for primary mucosal melanoma of the head and neck. Cancer.

[16] Owens JM, Roberts DB, Myers JN (2003). The role of postoperative adjuvant radiation therapy in the treatment of mucosal melanomas of the head and neck region. Arch Otolaryngol Head Neck Surg.

[17] Brandwein MS, Rothstein A, Lawson W, Bodian C, Urken ML (1997). Sinonasal melanoma. A clinicopathologic study of 25 cases and literature meta-analysis. Arch Otolaryngol Head Neck Surg.

[18] Cochrane Handbook for Systematic Reviews of Interventions. Available at http://handbook.cochrane.org/ Accessed 2014 Dec 06

[19] The Newcastle-Ottawa Scale [NOS] for assessing the quality of non-randomized studies in meta-analyses. Available at http://www.ohri.ca/programs/clinical_epidemiology/oxford.asp Accessed 2014 Dec 6

[20] Tierney JF, Stewart LA, Ghersi D, Burdett S, Sydes MR (2007). Practical methods for incorporating summary time-to-event data into meta-analysis. Trials.

[21] Meleti M, Leemans CR, de Bree R, Vescovi P, Sesenna E, van der Waal I (2008). Head and neck mucosal melanoma: experience with 42 patients, with emphasis on the role of postoperative radiotherapy. Head Neck.

[22] Sun CZ, Li QL, Hu ZD, Jiang YE, Song M, Yang AK (2014). Treatment and prognosis in sinonasal mucosal melanoma: a retrospective analysis of 65 patients from a single cancer center. Head Neck.

[23] Dauer EH, Lewis JE, Rohlinger AL, Weaver AL, Olsen KD (2008). Sinonasal melanoma: a clinicopathologic review of 61 cases. Otolaryngol Head Neck Surg.

[24] Leong SP, Accortt NA, Essner R, Ross M, Gershenwald JE, Pockaj B (2006). Impact of sentinel node status and other risk factors on the clinical outcome of head and neck melanoma patients. Arch Otolaryngol Head Neck Surg.

[25] Thaler ER, Kotapka M, Lanza DC, Kennedy DW (1999). Endoscopically assisted anterior cranial skull base resection of sinonasal tumors. Am J Rhinol.

[26] Keller DS, Thomay AA, Gaughan J, Olszanski A, Wu H, Berger AC, et al. Outcomes in patients with mucosal melanomas. J Surg Oncol. 2013. 10.1002/jso.23445 24132665

[27] Lourenco SV, Fernandes JD, Hsieh R, Coutinho-Camillo CM, Bologna S, Sangueza M (2014). Head and Neck Mucosal Melanoma: A Review. Am J Dermatopathol..

[28] Patel SG, Prasad ML, Escrig M, Singh B, Shaha AR, Kraus DH (2002). Primary mucosal malignant melanoma of the head and neck. Head Neck.

[29] Rinaldo A, Shaha AR, Patel SG, Ferlito A (2001). Primary mucosal melanoma of the nasal cavity and paranasal sinuses. Acta Otolaryngol.

[30] Mizumoto M, Hashii H, Senarita M, Sakai S, Wada T, Okumura T (2013). Proton beam therapy for malignancy in Bloom syndrome. Strahlenther Onkol.

[31] Douglas CM, Malik T, Swindell R, Lorrigan P, Slevin NJ, Homer JJ (2010). Mucosal melanoma of the head and neck: radiotherapy or surgery?. J Otolaryngol.

[32] Overgaard J (1986). The role of radiotherapy in recurrent and metastatic malignant melanoma: a clinical radiobiological study. Int J Radiat Oncol Biol Phys.

[33] Wada H, Nemoto K, Ogawa Y, Hareyama M, Yoshida H, Takamura A (2004). A multi-institutional retrospective analysis of external radiotherapy for mucosal melanoma of the head and neck in Northern Japan. Int J Radiat Oncol Biol Phys.

[34] Christopherson K, Malyapa RS, Werning JW, Morris CG, Kirwan J, Mendenhall WM (2015). Radiation Therapy for Mucosal Melanoma of the Head and Neck. Am J Clin Oncol..

[35] Madani I, Bonte K, Vakaet L, Boterberg T, De Neve W (2009). Intensity-modulated radiotherapy for sinonasal tumors: Ghent University Hospital update. Int J Radiat Oncol Biol Phys.

[36] Combs SE, Konkel S, Thilmann C, Debus J, Schulz-Ertner D (2007). Local high-dose radiotherapy and sparing of normal tissue using intensity-modulated radiotherapy (IMRT) for mucosal melanoma of the nasal cavity and paranasal sinuses. Strahlenther Onkol.

[37] Chan AW, Liebsch NJ (2008). Proton radiation therapy for head and neck cancer. J Surg Oncol.

[38] Rieken S, Habermehl D, Nikoghosyan A, Jensen A, Haberer T, Jakel O (2011). Assessment of early toxicity and response in patients treated with proton and carbon ion therapy at the Heidelberg ion therapy center using the raster scanning technique. Int J Radiat Oncol Biol Phys.

[39] Steneker M, Lomax A, Schneider U (2006). Intensity modulated photon and proton therapy for the treatment of head and neck tumors. Radiother Oncol.

[40] Zenda S, Kawashima M, Nishio T, Kohno R, Nihei K, Onozawa M (2011). Proton beam therapy as a nonsurgical approach to mucosal melanoma of the head and neck: a pilot study. Int J Radiat Oncol Biol Phys.

[41] Yanagi T, Mizoe JE, Hasegawa A, Takagi R, Bessho H, Onda T (2009). Mucosal malignant melanoma of the head and neck treated by carbon ion radiotherapy. Int J Radiat Oncol Biol Phys.

[42] Demizu Y, Fujii O, Terashima K, Mima M, Hashimoto N, Niwa Y (2014). Particle therapy for mucosal melanoma of the head and neck : A single-institution retrospective comparison of proton and carbon ion therapy. Strahlenther Onkol.

[43] Curtin JA, Fridlyand J, Kageshita T, Patel HN, Busam KJ, Kutzner H (2005). Distinct sets of genetic alterations in melanoma. N Engl J Med.

[44] Chapman PB, Hauschild A, Robert C, Haanen JB, Ascierto P, Larkin J (2011). Improved survival with vemurafenib in melanoma with BRAF V600E mutation. N Engl J Med.

[45] Carvajal RD, Antonescu CR, Wolchok JD, Chapman PB, Roman RA, Teitcher J (2011). KIT as a therapeutic target in metastatic melanoma. JAMA.

[46] Ascierto PA, Schadendorf D, Berking C, Agarwala SS, van Herpen CM, Queirolo P (2013). MEK162 for patients with advanced melanoma harbouring NRAS or Val600 BRAF mutations: a non-randomised, open-label phase 2 study. Lancet Oncol.

[47] Urri-Zanoni M, Medicina D, Lombardi D, Ungari M, Balzarini P, Rossini C (2013). Sinonasal mucosal melanoma: Molecular profile and therapeutic implications from a series of 32 cases. Head Neck.

[48] Schoenewolf NL, Bull C, Belloni B, Holzmann D, Tonolla S, Lang R (2012). Sinonasal, genital and acrolentiginous melanomas show distinct characteristics of KIT expression and mutations. Eur J Cancer.

[49] Cohen Y, Rosenbaum E, Begum S, Goldenberg D, Esche C, Lavie O (2004). Exon 15 BRAF mutations are uncommon in melanomas arising in nonsun-exposed sites. Clin Cancer Res.

[50] Rosenberg SA, Lotze MT, Yang JC, Topalian SL, Chang AE, Schwartzentruber DJ (1993). Prospective randomized trial of high-dose interleukin-2 alone or in conjunction with lymphokine-activated killer cells for the treatment of patients with advanced cancer. J Natl Cancer Inst.

[51] Kanetaka S, Tsukuda M, Takahashi M, Komatsu M, Niho T, Horiuchi C (2011). Mucosal melanoma of the head and neck. Exp Ther Med.

[52] Gavriel H, McArthur G, Sizeland A, Henderson M (2011). Review: mucosal melanoma of the head and neck. Melanoma Res.

[53] Mendenhall WM, Amdur RJ, Hinerman RW, Werning JW, Villaret DB, Mendenhall NP (2005). Head and neck mucosal melanoma. Am J Clin Oncol.

[54] Spugnini EP, Dragonetti E, Vincenzi B, Onori N, Citro G, Baldi A (2006). Pulse-mediated chemotherapy en hances local control and survival in a spontaneous canine model of primary mucosal melanoma. Melanoma Res.

[55] Tsukuda M, Mochimatsu I, Sakumoto M, Mikami Y, Yuyama S, Yanoma S (1993). The synergistic effects of interleukin 2 and interleukin 7 on the proliferation and autologous tumor cell lysis of tumor-associated lymphocytes. Biotherapy.

